# Effects of Apple Vinegar Addition on Aerobic Deterioration of Fermented High Moisture Maize Using Infrared Thermography as an Indicator

**DOI:** 10.3390/s22030771

**Published:** 2022-01-20

**Authors:** Aylin Agma Okur, Kerem Gozluklu, Ersen Okur, Berrin Okuyucu, Fisun Koc, Mehmet Levent Ozduven

**Affiliations:** 1 Department of Animal Science, Agricultural Faculty, Tekirdag Namik Kemal University, Tekirdag 59030, Turkey; keremgozluklu1992@gmail.com (K.G.); berrinokuyucu25@hotmail.com (B.O.); fkoc@nku.edu.tr (F.K.); lozduven@nku.edu.tr (M.L.O.); 2 Department of Biosystem Engineering, Agricultural Faculty, Tekirdag Namik Kemal University, Tekirdag 59030, Turkey; eokur@nku.edu.tr

**Keywords:** apple vinegar, sodium diacetate, high moisture maize grain, aerobic stability, infrared thermography

## Abstract

This study was carried out to determine the effects of apple vinegar and sodium diacetate addition on the aerobic stability of fermented high moisture maize grain (HMM) silage after opening. In the study, the effect of three different levels (0%, 0.5% and 1%) of apple vinegar (AV) and sodium diacetate (SDA) supplementation to fermented HMM at two different storage conditions (27–29 °C, 48% Humidity; 35–37 °C, 26% Humidity) were investigated. The material of the study was fermented rolled maize grain with 62% moisture content stored for about 120 days. Silage samples were subjected to aerobic stability test with three replicates for each treatment group. Wendee and microbiological analyses were made at 0, 2, 4, 7, and 12 days. Meanwhile, samples were displayed in the T200 IR brand thermal camera. According to the thermogram results, 1% SDA addition positively affected HMM silages at the second and fourth days of aerobic stability at both storage conditions (*p* < 0.05). Aerobic stability and infrared thermography analysis indicated that 1% AV, 0.5%, and 1% SDA additions to HMM silages had promising effects. Due to our results, we concluded that thermal camera images might be used as an alternative quality indicator for silages in laboratory conditions.

## 1. Introduction

Conservation of forage and cereal grains as silage is a very important, common source of ruminant nutrition around the world [1,2]. The silage process might be divided in four stages: (1) The aerobic stage in the silo immediately after harvest, (2) the fermentation stage, (3) the stable storage stage in the silo, and (4) the feed-out stage when the silo face is opened and exposed to air. Producing high-quality silage, and also avoiding dry matter losses as much as possible, is a challenge, and quality problems can occur any time during the entire silage process [3].

High moisture maize grain (HMM) is an indispensable part of the total mixed ration (TMR), especially in dairy cattle diets; TMR might comprise 18–20% HMM [4]. However, the high moisture and starch contents of HMM poses a significant risk in terms of its susceptibility to aerobic degradation during the feeding period [5,6,7,8,9]. However, to reduce the labor in field conditions, ensilaged HMM is taken from the silo in the amount that would not meet the daily requirements of animals but instead meet the requirements for 5–7 days. In addition, sometimes the ensilaged HMM is transferred from one farm to another to meet their needs. These situations lead to undesirable consequences in terms of aerobic deterioration. The objective of the study was to improve the aerobic stability of HMM silage by using additives that might be easily prepared and applied. In addition, to reveal the appropriate usage dosages and effects of the additives against the mentioned challenges.

A wide variety of silage additives have been used to preserve silage for decades. Apple vinegar and sodium diacetate can be identified as chemical additive groups that contain acids and their salts. Previous researches have established that sodium diacetate as an effective silage additive [10]. Apple vinegar (AV) consists of mainly acetic acid and is used as a natural food preserver for its antibacterial and antioxidant activities that are attributed to its organic acid content [11]. Besides, AV might be produced easily at home or farm conditions as an advantage.

The primary cause of silage quality deterioration is respiration. Even if ensiled material is not exposed to oxygen during the production, fermentation, and stable periods, it is an unavoidable condition when the silo is opened, and conditions turned to the aerobic stage with air entering the silo [12,13,14]. During the decomposition process, the dry matter breaks down into H_2_O and CO_2_ with a release of heat [15]. Aerobic deterioration occurs by the activities of aerobic microorganisms (yeasts, moulds, etc.), such as using water-soluble carbohydrates and fermentation products, and concluded with dry matter loss, energy release, increase in pH value and heat, as well as a decrease in protein and cellulose digestibilities [12,16]. In addition, growing moulds may produce mycotoxins, which threaten the health of humans and animals [17,18].

Silage temperature is proof of good silage management and subsequent handling. If it exceeds 20 °C even in the summer conditions, it would indicate that silage is not consumable for dairy cattle. Gálik et al. [14] also stated that heat increase up to 30 °C in maize silage could cause nutrition losses (1.7% of dry matter per day) due to anaerobic fermentation in silage. As a result of that, it would be useful to monitor heat differences and detect abnormalities in early stages. Thermal camera imaging might be used to serve this purpose.

Infrared thermal camera imaging is an objective, non-invasive quality evaluation tool to assess inflammatory reactions, early-stage mastitis, detections of ovarium cycles, foot pathologies in ruminants, equines, poultry, grain quality, as well as deterioration of feed and silage [19,20,21,22,23].

This study aims to evaluate the effects of apple vinegar and sodium diacetate addition on aerobic stability of high moisture fermented grain maize silage and also to reveal the changes of silage quality at different storage conditions by using temperature data logger and thermal camera images.

## 2. Materials and Methods

### 2.1. Silage Material

High moisture (62%) maize grain crushed and fermented in a plastic sausage silo for approximately 120 days was used as the research material. At the end of the silage fermentation process, approximately 40 kg of silage sample was brought to the laboratory. Samples from freshly opened silage were taken and analyzed to evaluate the initial material. Meanwhile, the fermented high moisture maize samples were then divided into 12 treatment groups with 3 replicates in each. Treatment groups consist of two different supplements (apple vinegar and sodium diacetate) with three different levels (0%, 0.5% and 1%) and stored at two different storage conditions (a room and incubator). Each treatment group was weighed as 1000 gr and put in plastic bags. Then additives were sprayed on the silage material and mixed for homogeneity. In the control groups, 20 mL of purified water were added as an equivalent dose to the treatment groups. After supplementation of additives, silage samples were stored for 12 days to evaluate the aerobic stability changes in room (27–29 °C; 48% humidity) and incubator (35–37 °C; 26% humidity) conditions. 

### 2.2. Laboratory Analysis

Dry matter (DM), pH, lactic acid (LA), water soluble carbohydrate (WSC), ammonia-nitrogen (NH3-N), lactic acid bacteria, yeast, and mould counts were determined at 0th, 2nd, 4th, 7th, and 12th days of storage to evaluate the aerobic stability of silage samples. Temperature changes in the storage ambient and inside the silage samples have been recorded by data logger devices (Hobo pendant, Bourne, MA, USA) every 30 min during the experiment period.

Chemical analyses were performed on triplicate samples. DM was determined by oven drying for 48 h at 60 °C. The pH in fresh material and silage samples was measured according to the British standard method [24]. The ammonia nitrogen (NH3-N) content of silages was determined, according to Jackson et al. [24]. The WSC content of silages was determined by spectrophotometer (Shimadzu UV-1201, Kyoto, Japan); after a reaction with the antron reagent [25]. Lactic acid (LA) was determined by the spectrophotometric method [26]. Microbiological evaluation included enumeration of lactobacilli on pour-plate Rogosa agar (Oxoid CM627, Oxoid, Basingstoke, UK). Yeast and moulds were determined by pour-plating in malt extract agar (Oxoid CM59) that had been acidified, after autoclaving, by the addition of 85% LA at a concentration of 0.5% *vol/vol*. Plates were incubated aerobically at 32 °C for 48–72 h.

### 2.3. Infrared Thermal Camera Imaging

Thermal camera imaging was recorded by using a Fluke Ti9 IR (IR Sensor Size: 160 × 120 Focal Plane Array, WA, USA) thermal camera from a 1-m distance with a surface size of 20 × 30 cm (with two replicates from each sample; *n* = 24). Thermography images were taken in laboratory conditions (ambient temperature: 22 °C) without direct sunlight and air velocity. The average, minimum, and maximum temperatures of the silage surface were calculated using SmartView^®^ software program in which each pixel of the image was allocated to one temperature value.

### 2.4. Statistical Analysis

The research was conducted according to the 2 × 2 × 3 factorial trial design. To reveal the effects of treatment and temperature, the data were evaluated according to the variance analysis technique. If the difference between groups was found to be significant, Duncan’s range test was applied [27]. The applied mathematical model was as follows (Equation (1)):

Yijk = µ + Ai + Bj+ Ck + (AB)ij + (AC)ik + (BC)jk + (ABC)ijk + eijkl.(1)

Yijk: Observation applying the i^th^ supplement with j^th^ inclusion level and stored at k^th^ temperature’

µ: Overall average;

Ai: Effect of i^th^ supplement;

Bj: Effect of j^th^ inclusion level of supplement;

Ck: Effect of k^th^ storage temperature;

(AB)ij: Interaction effect of i^th^ supplement × j^th^ inclusion level;

(AC)ik: Interaction effect of i^th^ supplement × k^th^ storage temperature;

(BC)jk: Interaction effect of j^th^ inclusion level × k^th^ storage temperature;

(ABC)ijk: Interaction effect of i^th^ supplement × j^th^ inclusion level × k^th^ storage temperature;

eijkl: Error associated with each observation.

## 3. Results and Discussion

This section may be divided by subheadings. It should provide a concise and precise description of the experimental results, their interpretation, as well as the experimental conclusions that can be drawn.

In Table 1, the analysis results of the beginning material of fermented maize silage are given. Mould has not been detected in the initial materials. As a result of thermal camera footage carried out on day zero of aerobic stability, the average temperature of fermented maize silage was 30.62 °C, while the background temperature was 22 °C.

Demirel et al. [28] stated that there is an inverse relationship between the DM and pH levels of the raw material to be ensilaged. Their results showed that the pH value of corn silage (with 23.48% DM) harvested during the milking phase was 4.15. The pH value of HMM silage was found to be low in the intial material (such as 3.900). These results might be evaluated as a harmonious result with Demirel et al. [28].

### 3.1. 2nd Day of Aerobic Deterioration

Second-day aerobic stability results of HMM silages are presented in Table 2. In the LAB count results, the lowest value was observed in the groups that has 0.5% SDA added and were stored in the incubator, and the highest value was observed in the groups that had 1% AV added and were stored at room temperature. In addition, significant reductions in LAB numbers were observed in all silage samples stored in the incubator (35–37 °C, 26% humidity; *p* < 0.001). High temperatures with low humidity values might be responsible for the change in LAB counts. However, no yeast could be detected in groups with 0% and 1% AV and 1% SDA supplemented and stored in room conditions (*p* < 0.001). According to the mould count results, the highest value was found in a SDA control group stored in incubator conditions with 3.310 log_10_ cfu/g, while the lowest value was 2.220 log_10_ cfu/g in 0.5% vinegar added and stored under room conditions (*p* < 0.001).

### 3.2. 4th Day of Aerobic Deterioration

Aerobic stability analysis results of HMM silage on the fourth day of storage were given at Table 3. LAB counts were intended to be lower at SDA added groups and also lower at groups stored at 35–37 °C (*p* < 0.001). In addition, the highest LA level was observed at 0.5% SDA supplemented and stored at room conditions (27–29 °C; 48% Humidity; *p* < 0.05). In addition, the lowest yeast counts were observed at 0.5 and 1% SDA added and stored at high temperatures (*p* < 0.001). However, WSC levels were not affected by additive sources (*p* > 0.05). In order to avoid the deterioration of the ensilaged material, there must be LAB in the silage and also sufficient amount of WSC. Thus, LAB can produce LA required for silage fermentation by using WSC in the medium [29]. However, Alçiçek and Özkan [30] reported that LA content should not be higher than 2% for good silage quality.

Fermentation properties of silages are also effective on aerobic deterioration. Unused sugars and high levels of LA in the silage reduce aerobic stability. Some yeasts and moulds might cause CO_2_ production in silages by using the remaining sugars and LA as nutrients. As a result, an increase occurs in ambient pH and temperature [31]. The data obtained from the study support the previous research results [32,33,34].

### 3.3. 7th Day of Aerobic Deterioration

The effects of additives on HMM silage on the seventh day of aerobic stability are presented in Table 4. When pH, NH3-N, WSC analysis results, LAB, and yeast numbers are evaluated, it has seen that the highest values belong to the SDA control group under room conditions (27–29 °C; 48% Humidity; *p* < 0.001). However, the highest LA value (13.740 g/kg DM) was found in silages stored in the incubator with 0.5% SDA, while the lowest LA (1.147 g/kg) was observed in silages stored in room conditions with 1% AV addition (*p* < 0.001). The effects of the silage additive, addition level, storage temperature, their double and triple interactions on pH, NH3-N, LA, WSC, and LAB values were found to be statistically different (*p* < 0.001).

Pahlow et al. [17] reported that the number of yeast in high-moisture maize silages was 3–5 log_10_ cfu/g and that a high yeast count reduced aerobic stability, especially at high temperatures. Teller et al. [35] stated that physical damage to the grain might cause substrate formation for microorganisms and might cause the high yeast content in high-moisture maize silages. Considering the results of the research, the high yeast content in the control group, particularly at high temperatures supports previous studies on this subject. Besides, there was a decrease in the yeast and LAB numbers of silages stored in the incubator. In addition, mould counts were found to be zero in silages stored in incubator conditions for all additives, and additional levels (*p* < 0.001). In the study, silage samples stored at 35–37 °C had relatively low humidity (26%). That might have affected microbial growth negatively. However, a 1% SDA addition has been found effective in both room and incubator conditions on LAB and Yeast counts.

### 3.4. 12th Day of Aerobic Deterioration

The 12th day analysis results of aerobic stability are given in Table 5. According to the pH results, it was observed that the lowest values were found in silages stored at high temperatures and the effect of storage temperatures was statistically significant (*p* < 0.001). Similarly, a decrease was observed in NH3-N values under high temperature storage conditions (*p* < 0.01). However, the NH3-N value was found to be lower in the group containing 1% SDA and stored in room conditions (*p* < 0.01).

In addition, WSC contents were found to be lower in all groups stored at room conditions, too. However, one of the lowest values were detected at the 0.5%-AV added and incubator-stored group. McDonald [15] has stated that WSC are the most important energy source used by lactic acid bacteria. Some researchers observed an increase in the amount of WSC due to increasing SDA supplementation. Researchers attributed this increase to the antifungal properties of additives [36,37,38]. They prevent the growth of unwanted microorganisms and caused a reduction of DM and nutrition losses. In the study, all parameters (additives, addition ratios, and storage conditions) revealed significant effects on WSC (*p* < 0.001).

LA levels demonstrated an increase at higher storage temperatures except for 1.0% SDA-added groups. Besides, the highest value (11.407 g/kg) was determined in the 1% AV-added groups. Reeves et al. [39] reported that the amount of LA in dry matter of maize silage varied between 1.58% and 8.57%. In addition, Deswysen et al. [40] reported that the amount of lactic acid on dry matter in maize silage was 6.31%, while Phillip and Hidalgo [41] reported that it was 5%. In the present study, LA levels ranged between 0.881–1.469% on the second day, and 0.030–1.141% on the 12th day of aerobic stability (Table 2 and Table 5).

According to the results of the 12th day of aerobic stability, there was an increase in LAB counts of silage samples stored in the incubator compared to those in the chamber, but a decrease in yeast count was observed (except vinegar control and 0.5% SDA supplemented groups) (*p* < 0.001). In addition to these results, mould counts were found to be 0 in all treatment groups on the 12th day of aerobic stability.

### 3.5. Thermal Camera Imaging Results of Aerobic Stability

**Figure 1:**
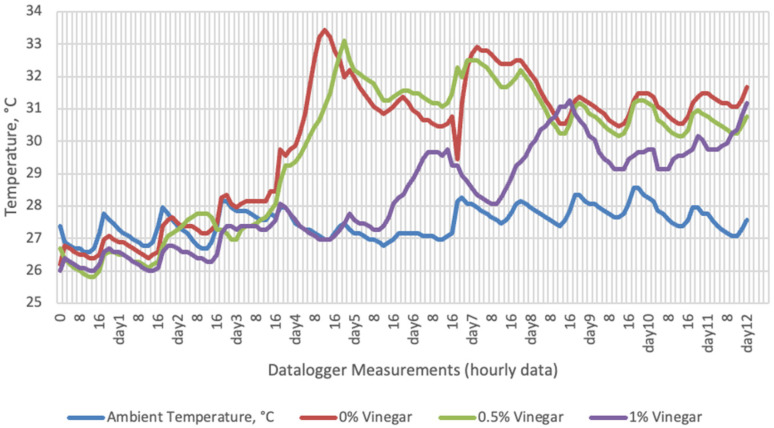
Temperature changes of apple vinegar supplemented groups stored at room conditions for 12 days (Ambient temperatures of room conditions: 27–29 °C; 48% Humidity).

**Figure 2:**
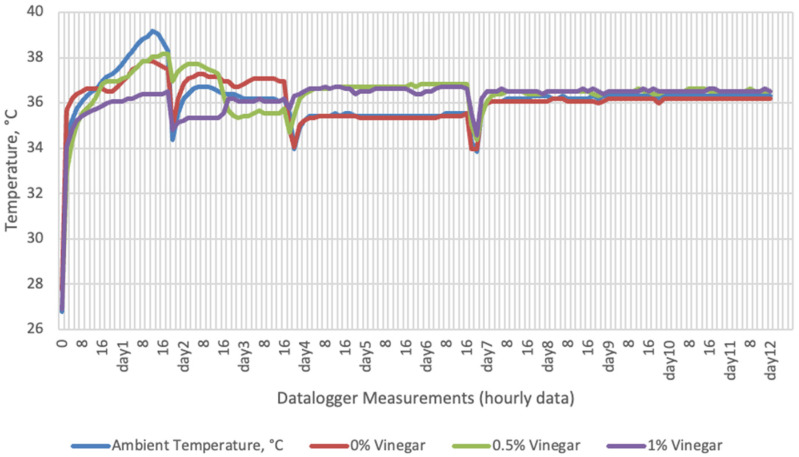
Temperature changes of apple vinegar supplemented groups stored at incubator conditions for 12 days (Ambient temperatures of incubator conditions: 35–37 °C; 26% Humidity).

**Figure 3:**
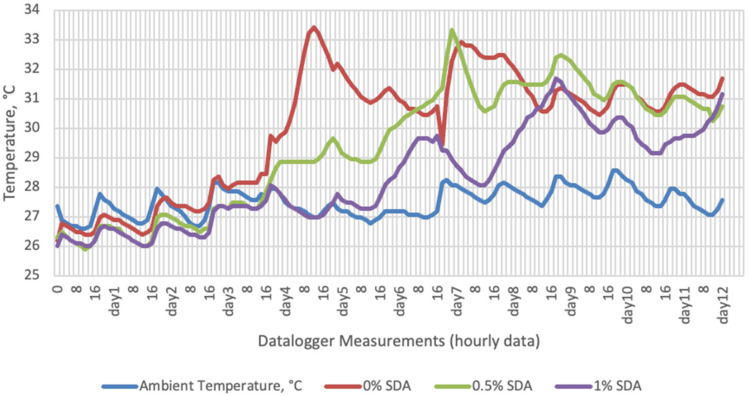
Temperature changes of SDA-supplemented groups stored at room conditions for 12 days (Ambient temperatures of room conditions: 27–29 °C; 48% Humidity).

**Figure 4:**
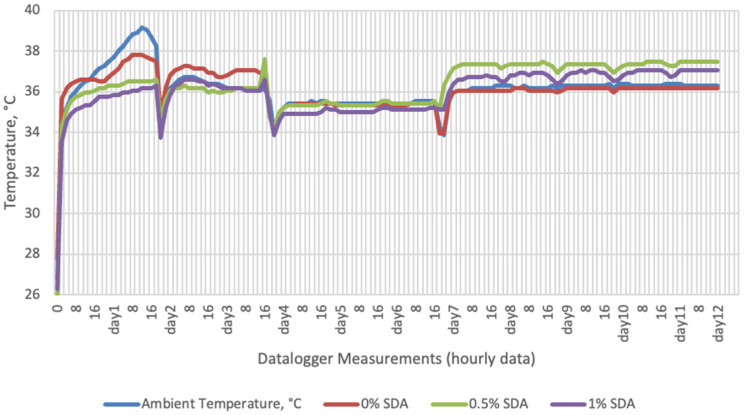
Temperature changes of SDA supplemented groups stored at incubator conditions for 12 days (Ambient temperatures of incubator conditions: 35–37 °C; 26% Humidity).

Temperature data obtained from dataloggers for 12 days were summarized in Figure 1, Figure 2, Figure 3 and Figure 4. The temperatures of sensor data and thermal camera images in the study showed compatible results with each other. The finding was parallel with two field studies [42,43].

**Figure 5:**
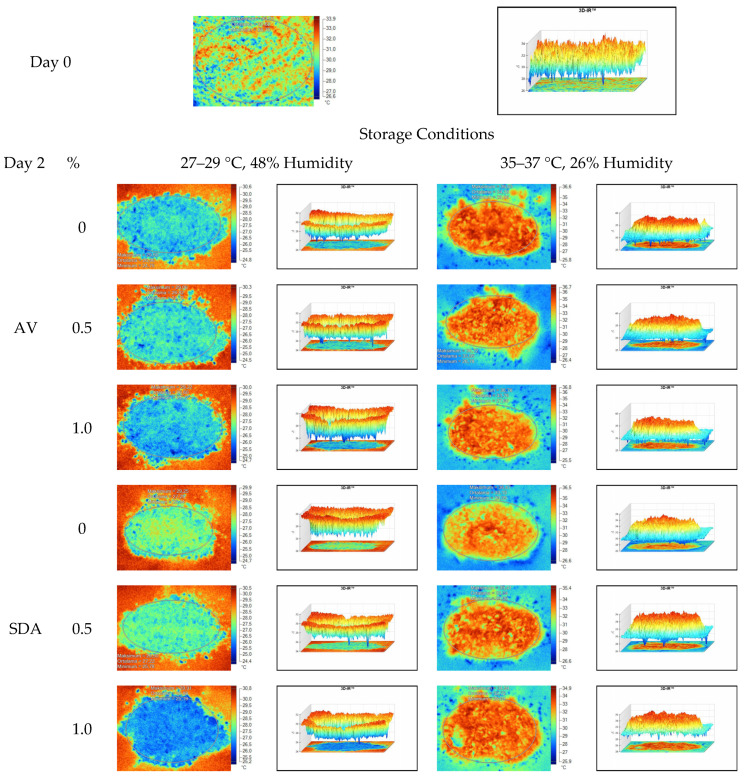
Thermal camera imaging samples of aerobic stability on day 2 (Ambient temperature = 22 °C; AV: Apple vinegar; SDA: Sodium diacetate).

**Figure 6:**
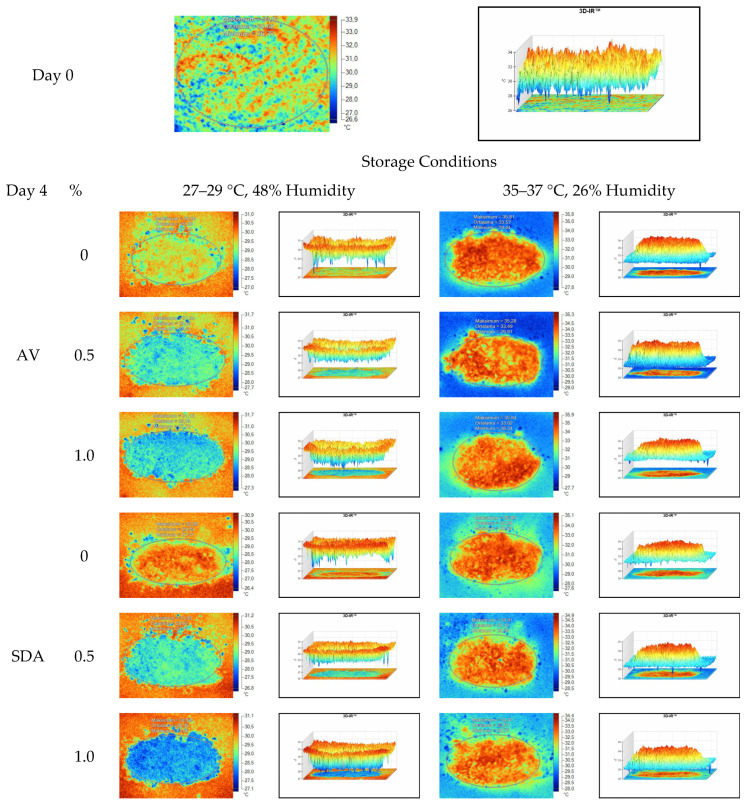
Thermal camera imaging samples of aerobic stability on day 4 (Ambient temperature = 22 °C; AV: Apple vinegar; SDA: Sodium diacetate).

**Figure 7:**
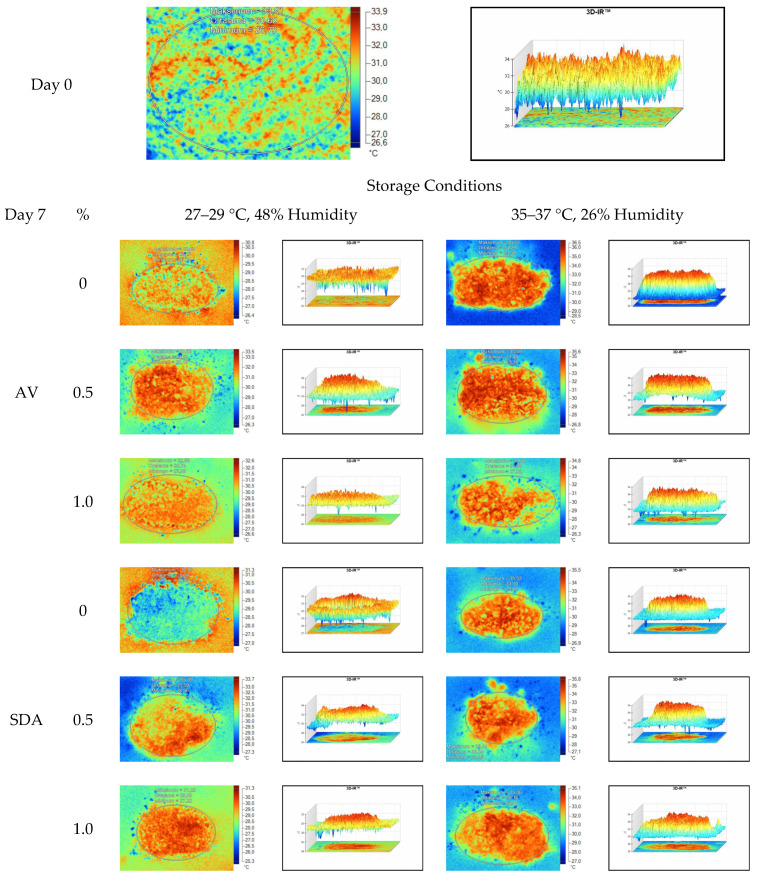
Thermal camera imaging samples of aerobic stability on day 7 (Ambient temperature = 22 °C; AV: Apple vinegar; SDA: Sodium diacetate).

**Figure 8:**
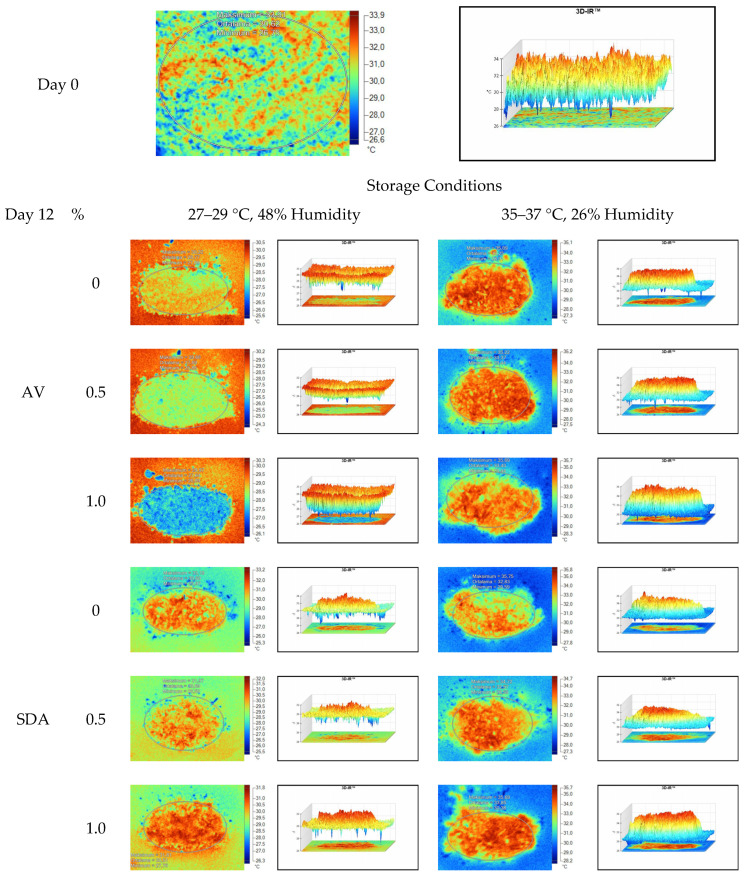
Thermal camera imaging samples of aerobic stability on day 12 (Ambient temperature = 22 °C; AV: Apple vinegar; SDA: Sodium diacetate).

Mean, maximum, minimum, and standard deviation values of temperature measurements of aerobic stability period were determined by thermal camera with the results presented in Table 6, Table 7, Table 8 and Table 9. Arithmetic means was subsequently created on the basis of all values. Data logger records at the time of thermograms taken were also given in the tables. Additionally, one of the thermograms captured from each treatment (*n* = 12; same samples during the experiment) are shown at Figure 5, Figure 6, Figure 7 and Figure 8. They might help to visualize the heat differences in the silage samples.

On the 2nd and 4th day of aerobic stability, the lowest temperature values were observed in the 1% SDA added HMM silages at both storage temperatures (Table 6 and Table 7). In the thermal camera measurements conducted on the 7th day of storage, the lowest temperature values were observed in the SDA control group kept under room conditions (Table 8). Thermograms of the 12th day of aerobic stability showed that the lowest values were found in the groups that had AV added and were kept under room conditions (Table 9). These results supported the importance of storage conditions and additives on quality properties of silages even after opening (*p* < 0.01).

Thermal camera imaging results of aerobic stability approved that the temperature of silage samples stored in 35–37 °C was higher compared to the stored at 27–29 °C. However, when the temperature differences between the silages and storage environment were considered, it is seen that the temperature differences increase in the room conditions (27–29 °C; 48% humidity), heat rises in the samples, and therefore deterioration occurs in stored silages. Similarly Kaya and Polat [44] reported that more than 2 °C of the temperature difference between the ambient and silage may indicate deterioration. Koc et al. [45] also indicated that heat differences are major signs of deterioration.

Heat differences in maize silages stored at 27–29 °C, started to increase at day 4. However, on day 7 differences had maximized and started to decline at day 12. Due to this increase on the seventh day, pH values, yeast, and mould counts were found higher for all treatments in room conditions. Similarly, Junga and Trávníček [13] have found a connection between infrared thermography images with chemical, and microbial analysis results in the field conditions. Samples from surfaces with a higher temperature have resulted with an increase in pH values, mould, and yeast counts. Santos et al. [18] indicated that feeding silages with high concentrations of yeasts from aerobic spoilage is often implicated as a cause of poor animal performance on dairy animals.

Borreani and Tabacco [46] evaluated the temperature at 11 locations on 54 silos and correlated the temperature with chemical composition and microbial count. They also concluded that temperature is linked to microbial activity and might be an important indicator of the early stages of aerobic degradation.

Addah et al. [21] used an infrared thermal imaging technique to assess that the heating of barley silages stored in large cylindrical bag silos could serve as a simple and rapid method of directly measuring and visualizing heat distribution over the feed-out face of silos in real-time.

Alsaaod et al. [23] reported that environmental factors such as airflow, environment temperature, humidity, sunlight, and motion could be affected by the thermograms. The preferred environment conditions appear to be in the neutral zone temperature, without direct sunlight and detectable airflow. In addition, infrared thermal imaging technique does not need light or shadow to capture the frame accurately [47].

Researchers suggested that infrared thermography might be used for quick temperature detection of silo surface, of silage layers, and also might be a practical method for assessing the aerobic stability of silages on field and laboratory conditions [13,21,43,48]. According to the results of study, thermal camera imaging technique might be used to detect temperature differences and to reveal deterioration in the silage.

## 4. Conclusions

Fermented high moisture maize grain (HMM) is an important part of the TMR for dairy cattle rations with 18–20% inclusion levels. However, low dry matter and high starch contents of HMM increase aerobic deterioration risks during the feeding period. The objective of the study was to improve and reveal aerobic stability duration after opening HMM silage by easily prepared and supplemented additives.

In the light of the study results, it was seen that the silage additives and their usage levels had a positive effect on dry matter, NH3-N, LA production, WSC amount, LAB, and yeast counts. In addition to these results, it was observed that the storage temperature also had a significant effect on pH, NH3-N, LA production, WSC, the numbers of LAB, and yeast. The additive usage (%) and their effects on aerobic stability parameters were not in line with the study. Differences in this regard may be due to the chemical, physical, and microbiological properties of the beginning material.

When the thermal camera images were evaluated, the temperature differences between the silage and storage environment might be able to become more visible. Due to the increase in temperature differences at room conditions on the fourth day of aerobic stability, deterioration was observed in HMM silage. The similarity and relation between chemical analysis and microbial composition results are also noteworthy. The results support that the infrared thermography method might be an effective tool in the early detection of silage deterioration in laboratory conditions, by determining temperature differences and heat spots in silage.

It was concluded that supplementing different doses of apple vinegar and sodium diacetate to HMM silages after silo opening improved aerobic stability. However, further studies were needed to determine effective dosages of apple vinegar on field conditions. and also requires support with in vivo studies for feed palatability.

## Figures and Tables

**Table 1:** Chemical and microbiological analysis results for the beginning material (day 0).

Parameters *	Analysis Results
pH	3.900
DM, % FM	62.021
NH3-N g/kg DM	1.289
LA, g/kg DM	9.200
WSC, g/kg DM	11.418
LAB, cfu/g DM	2.712
Yeast, cfu/g DM	2.723
Mould, cfu/g DM	0

* DM: Dry matter, FM: Fresh material, NH3-N: Ammonia-nitrogen, LA: Lactic acid, WSC: Water-soluble carbohydrate, LAB: Lactic acid bacteria, and cfu: Colony forming unit.

**Table 2:** Effects of additives and storage conditions on the second day of aerobic stability of high moisture maize silage.

Additive	Usage %	Storage Temperature *	pH	DM%	NH3-N g/kg DM	LA g/kg DM	WSC g/kg DM	LAB log_10_ cfu/g	Yeast log_10_ cfu/g	Mould log_10_ cfu/g
AV	0	27–29 °C	3.850 c	59.550 b	1.577 ab	9.760 defg	9.870 g	4.303 bc	0.000 g	2.527 f
		35–37 °C	3.900 c	63.890 a	1.380 cde	13.600 ab	19.857 b	2.933 e	4.517 e	2.847 de
	0.5	27–29 °C	3.900 c	60.550 b	1.263 e	9.470 efg	17.440 de	4.533 ab	4.660 d	2.220 h
		35–37 °C	3.900 c	60.310 b	1.520 abc	11.473 bcdef	15.370 f	3.180 e	4.467 e	3.093 b
	1.0	27–29 °C	3.900 c	61.233 b	1.570 ab	8.810 g	19.613 bc	4.610 a	0.000 g	2.363 g
		35–37 °C	3.900 c	59.913 b	1.613 ab	11.790 bcde	18.043 d	4.053 cd	4.250 f	2.823 e
SDA	0	27–29 °C	3.850 c	60.140 b	1.330 de	9.280 fg	10.610 g	4.407 ab	4.830 c	2.993 bcd
		35–37 °C	3.900 c	58.760 c	1.687 a	14.690 a	17.530 d	2.920 e	4.780 c	3.310 a
	0.5	27–29 °C	4.007 b	60.673 b	1.470 bcd	10.753 cdefg	18.977 c	4.460 ab	4.777 c	3.030 bc
		35–37 °C	4.010 b	59.240 b	1.447 bcd	12.287 bc	16.667 e	2.227 f	4.903 b	2.890 cde
	1.0	27–29 °C	4.007 b	59.587 b	1.390 cde	12.047 bcd	15.420 f	3.933 d	0.000 g	3.000 bcd
		35–37 °C	4.200 a	59.870 b	1.540 abc	9.560 efg	20.750 a	3.050 e	5.070 a	2.913 cde
SEM			0.017	0.260	0.024	0.347	0.561	0.133	0.345	0.052
*p* values										
Additive			0.000	0.003	0.749	0.157	0.801	0.000	0.000	0.000
Usage			0.000	0.568	0.036	0.062	0.000	0.000	0.000	0.001
Temperature			0.000	0.910	0.004	0.000	0.000	0.000	0.000	0.000
Additive × Usage			0.000	0.125	0.041	0.762	0.000	0.000	0.000	0.073
Additive × Temperature			0.011	0.023	0.052	0.099	0.001	0.000	0.000	0.000
Usage × Temperature			0.012	0.032	0.890	0.001	0.000	0.000	0.000	0.037
Additive × Usage × Temperature			0.003	0.002	0.000	0.007	0.000	0.021	0.000	0.000

a–h: Values shown in different letters in the same column are statistically important (*p* < 0.05). * Room conditions: 27–29 °C, 48% Humidity. Incubator conditions: 35–37 °C, 26% Humidity. AV: Apple vinegar; SDA: Sodium diacetate; SEM: Standard error of means; DM: Dry matter; NH3-N: Ammonia-nitrogen, LA: Lactic acid; WSC: Water-soluble carbohydrate; and LAB: Lactic acid bacteria.

**Table 3:** Effects of additives and storage conditions on the fourth day of aerobic stability of high moisture maize silage.

Additive	Usage %	Storage Temperature *	pH	DM %	NH3-N g/kg DM	LA g/kg DM	WSC g/kg DM	LAB log_10_ cfu/g	Yeast log_10_ cfu/g	Mould log_10_ cfu/g
AV	0	27–29 °C	4.100 d	63.457 e	1.410 e	9.867 d	9.550 e	4.400 c	4.743 de	2.850 a
		35–37 °C	4.150 d	61.183 f	1.040 g	11.737 c	8.000 g	3.793 d	6.143 a	0.000 c
	0.5	27–29 °C	4.350 b	63.080 de	1.430 de	6.800 i	6.357 h	5.313 a	5.167 c	2.350 b
		35–37 °C	4.200 c	65.380 bc	1.267 f	9.250 e	8.900 f	4.800 b	5.800 b	0.000 c
	1.0	27–29 °C	4.100 d	63.383 de	1.547 bc	9.227 e	18.767 a	5.210 a	5.157 c	0.000 c
		35–37 °C	3.900 g	65.457 bc	1.580 b	7.710 h	12.300 c	3.760 d	3.950 f	0.000 c
SDA	0	27–29 °C	4.650 a	62.853 de	1.357 e	8.477 f	4.807 i	2.813 e	3.047 g	0.000 c
		35–37 °C	4.100 d	67.137 a	1.387 e	12.417 b	14.060 b	2.947 e	4.700 de	0.000 c
	0.5	27–29 °C	3.950 f	63.907 cd	1.540 bc	13.417 a	14.197 b	3.883 d	4.987 cd	0.000 c
		35–37 °C	4.000 f	62.050 ef	1.727 a	8.687 f	10.910 d	2.607 ef	2.700 h	0.000 c
	1.0	27–29 °C	4.100 d	63.733 d	1.240 f	9.327 e	8.897 f	2.310 f	4.530 e	0.000 c
		35–37 °C	4.000 f	66.463 ab	1.490 cd	8.037 g	11.100 d	2.680 e	2.770 h	0.000 c
SEM			0.033	0.311	0.030	0.322	0.619	0.172	0.186	0.165
*p* values										
Additive			1.000	0.025	0.000	0.000	0.884	0.000	0.000	0.000
Usage			0.000	0.005	0.000	0.000	0.000	0.000	0.000	0.000
Temperature			0.000	0.000	0.683	0.018	0.000	0.000	0.000	0.000
Additive × Usage			0.000	0.000	0.000	0.000	0.000	0.000	0.000	0.000
Additive × Temperature			0.000	0.094	0.000	0.000	0.000	0.000	0.000	0.000
Usage × Temperature			0.000	0.018	0.000	0.000	0.000	0.002	0.000	0.000
Additive × Usage × Temperature			0.000	0.000	0.029	0.000	0.000	0.000	0.000	0.000

a–i: Values shown in different letters in the same column are statistically important (*p* < 0.05). * Room conditions: 27–29 °C, 48% Humidity. Incubator conditions: 35–37 °C, 26% Humidity. AV: Apple vinegar; SDA: Sodium diacetate; SEM: Standard error of means; DM: Dry matter; NH3-N: Ammonia-nitrogen, LA: Lactic acid; WSC: Water-soluble carbohydrate; and LAB: Lactic acid bacteria.

**Table 4:** Effects of additives and storage conditions on the seventh day of aerobic stability of high moisture maize silage.

Additive	Usage %	Storage Temperature *	pH	DM %	NH3-N g/kg DM	LA g/kg DM	WSC g/kg DM	LAB log_10_ cfu/g	Yeast log_10_ cfu/g	Mould log_10_ cfu/g
AV	0	27–29 °C	6.000 c	72.757 a	1.837 b	1.720 g	16.097 b	5.560 ab	5.140 c	3.180 a
		35–37 °C	3.900 g	63.910 d	1.607 d	8.420 f	9.107 e	0.000 j	3.780 de	0.000 d
	0.5	27–29 °C	6.500 ab	63.203 d	1.617 d	1.680 g	7.737 f	2.980 h	5.690 b	2.280 b
		35–37 °C	3.900 g	61.950 ef	1.420 g	9.097 e	7.470 f	3.337 g	3.680 e	0.000 d
	1.0	27–29 °C	6.450 b	65.393 c	1.477 f	1.147 h	6.800 g	5.500 b	5.010 c	2.660 ab
		35–37 °C	3.900 g	63.140 de	1.557 e	8.480 f	8.937 e	4.193 d	4.027 d	0.000 d
SDA	0	27–29 °C	6.550 a	65.670 bc	1.897 a	9.917 c	18.207 a	5.627 a	6.160 a	1.460 c
		35–37 °C	3.950 fg	66.810 b	1.527 e	9.337 d	11.077 d	2.770 i	3.920 de	0.000 d
	0.5	27–29 °C	5.250 d	61.683 f	1.697 c	8.380 f	5.480 h	4.930 c	5.840 b	2.440 ab
		35–37 °C	4.000 f	62.223 ef	1.540 e	13.740 a	12.800 c	3.407 fg	3.930 de	0.000 d
	1.0	27–29 °C	4.250 e	59.837 g	1.817 b	10.267 b	7.617 f	3.927 e	3.883 de	2.480 ab
		35–37 °C	4.300 e	66.463 bc	1.527 e	9.367 d	8.737 e	3.470 f	4.023 d	0.000 d
SEM			0.186	0.553	0.025	0.640	0.628	0.256	0.150	0.221
*p* values										
Additive			0.000	0.000	0.000	0.000	0.000	0.000	0.212	0.050
Usage			0.000	0.000	0.000	0.000	0.000	0.000	0.000	0.741
Temperature			0.000	0.007	0.000	0.000	0.000	0.000	0.000	0.000
Additive × Usage			0.000	0.043	0.000	0.000	0.000	0.000	0.000	0.027
Additive × Temperature			0.000	0.000	0.000	0.000	0.000	0.000	0.316	0.050
Usage × Temperature			0.000	0.000	0.000	0.000	0.000	0.000	0.000	0.741
Additive × Usage × Temperature			0.000	0.000	0.000	0.000	0.000	0.000	0.000	0.027

a–j: Values shown in different letters in the same column are statistically important (*p* < 0.05). * Room conditions: 27–29 °C, 48% Humidity. Incubator conditions: 35–37 °C, 26% Humidity. AV: Apple vinegar; SDA: Sodium diacetate; SEM: Standard error of means; DM: Dry matter; NH3-N: Ammonia-nitrogen, LA: Lactic acid; WSC: Water-soluble carbohydrate; and LAB: Lactic acid bacteria.

**Table 5:** Effects of additives and storage conditions on the 12th day of aerobic stability of high moisture maize silage.

Additive	Usage %	Storage Temperature *	pH	DM %	NH3-N g/kg DM	LA g/kg DM	WSC g/kg DM	LAB log_10_ cfu/g	Yeast log_10_ cfu/g	Mould log_10_ cfu/g
AV	0	27–29 °C	6.900 b	71.577 a	2.693 a	0.757 e	16.517 d	0.000 i	3.377 f	0.000
		35–37 °C	3.950 d	67.417 abc	0.987 ef	6.677 d	55.230 b	3.547 b	5.670 b	0.000
	0.5	27–29 °C	7.800 a	57.083 d	2.600 a	0.370 e	17.827 d	2.613 f	6.330 a	0.000
		35–37 °C	3.900 d	66.283 bc	1.323 d	9.750 b	9.100 e	2.840 e	4.280 cd	0.000
	1.0	27–29 °C	7.450 ab	60.637 d	2.883 a	0.303 e	18.177 d	2.483 f	6.100 a	0.000
		35–37 °C	3.900 d	69.970 ab	1.237 de	11.407 a	67.757 a	3.477 b	4.390 cd	0.000
SDA	0	27–29 °C	6.950 b	70.547 ab	2.807 a	1.060 e	7.747 e	2.103 h	6.367 a	0.000
		35–37 °C	3.900 d	66.610 bc	1.363 cd	7.930 c	46.140 c	3.297 cd	3.797 e	0.000
	0.5	27–29 °C	7.200 ab	67.990 abc	2.250 b	0.837 e	15.760 d	3.410 bc	3.710 e	0.000
		35–37 °C	3.900 d	66.907 bc	1.433 cd	6.520 d	68.083 a	4.550 a	4.320 cd	0.000
	1.0	27–29 °C	6.200 c	65.157 c	0.890 f	9.020 b	19.190 d	2.273 g	4.607 c	0.000
		35–37 °C	4.050 d	65.157 c	1.677 c	6.840 d	55.610 b	3.240 d	4.247 d	0.000
SEM			0.278	0.732	0.124	0.679	3.800	0.180	0.174	-
*p* values										
Additive			0.041	0.050	0.002	0.012	0.000	0.000	0.000	-
Usage			0.138	0.000	0.002	0.000	0.000	0.000	0.063	-
Temperature			0.000	0.051	0.000	0.000	0.000	0.000	0.000	-
Additive × Usage			0.249	0.003	0.000	0.000	0.000	0.000	0.000	-
Additive × Temperature			0.023	0.000	0.000	0.000	0.000	0.000	0.029	-
Usage × Temperature			0.065	0.000	0.000	0.000	0.000	0.000	0.000	-
Additive × Usage × Temperature			0.085	0.017	0.000	0.000	0.000	0.000	0.000	-

a–i: Values shown in different letters in the same column are statistically important (*p* < 0.05). * Room conditions: 27–29 °C, 48% Humidity. Incubator conditions: 35–37 °C, 26% Humidity. AV: Apple vinegar; SDA: Sodium diacetate; SEM: Standard error of means; DM: Dry matter; NH3-N: Ammonia-nitrogen, LA: Lactic acid; WSC: Water-soluble carbohydrate; and LAB: Lactic acid bacteria.

**Table 6:** Thermal camera imaging results of aerobic stability on day 2 (Ambient temperature = 22 °C).

Aerobic Stability	Additive	%	Storage Temperature *	Thermal Camera Imaging Measurements, °C			
				Mean	Min	Max	Std. Dev.
Day 0	-	-	-	30.62	26.75	33.91	0.94
Day 2	AV	0	27.370 °C	27.280 de	25.280 b	29.845 d	0.420 c
			36.187 °C	33.945 ab	28.345 a	36.330 ab	1.205 a
		0.5	27.370 °C	26.945 de	24.595 b	29.515 d	0.490 c
			36.187 °C	34.360 a	27.935 a	36.925 a	1.225 a
		1.0	27.370 °C	26.865 de	24.720 b	29.640 d	0.475 c
			36.187 °C	33.740 ab	28.220 a	36.530 ab	1.225 a
	SDA	0	27.370 °C	27.435 d	25.610 b	29.375 d	0.435 c
			36.187 °C	33.555 ab	28.750 a	36.595 ab	1.225 a
		0.5	27.370 °C	27.110 de	25.530 b	29.280 d	0.450 c
			36.187 °C	33.100 bc	28.280 a	35.580 bc	1.070 ab
		1.0	27.370 °C	26.430 e	24.690 b	29.330 d	0.490 c
			36.187 °C	32.420 c	27.955 a	34.985 c	0.940 b
SEM				0.689	0.358	0.705	0.075
*p* values							
Additive				0.007	0.378	0.005	0.080
Usage				0.010	0.313	0.206	0.650
Temperature				0.000	0.000	0.000	0.000
Additive × Usage				0.188	0.594	0.172	0.263
Additive × Temperature				0.011	0.697	0.158	0.093
Usage × Temperature				0.694	0.896	0.340	0.140
Additive × Usage × Temperature				0.538	0.911	0.077	0.291

a–e: Values shown in different letters in the same column are statistically important (*p* < 0.05). * Storage temperatures were recorded by data logger on day 2, while thermal camera images were taken. AV: Apple vinegar; SDA: Sodium diacetate; Std. Dev.: Standard deviation; and SEM: Standard error of means.

**Table 7:** Thermal camera imaging results of aerobic stability on day 4 (Ambient temperature = 22 °C).

Aerobic Stability	Additive	%	Storage Temperature *	Thermal Camera Imaging Measurements, °C			
				Mean	Min	Max	Std. Dev.
Day 0	-	-	-	30.62	26.75	33.91	0.94
Day 4	AV	0	27.468 °C	29.530 bc	27.470 cd	31.000 c	0.365 d
			35.222 °C	33.575 a	29.345 a	35.655 a	1.115 a
		0.5	27.468 °C	29.535 bc	27.925 cd	31.250 c	0.380 d
			35.222 °C	33.515 a	29.565 a	35.390 ab	0.940 b
		1.0	27.468 °C	29.090 cd	27.265 cd	30.955 c	0.415 d
			35.222 °C	33.055 a	28.170 bc	35.265 ab	1.050 ab
	SDA	0	27.468 °C	29.995 b	27.375 cd	31.300 c	0.490 d
			35.222 °C	33.525 a	28.955 ab	35.450 ab	0.965 b
		0.5	27.468 °C	28.775 cd	27.345 cd	31.000 c	0.475 d
			35.222 °C	32.745 a	29.185 a	34.660 b	0.785 c
		1.0	27.468 °C	28.425 d	27.060 d	30.595 c	0.420 d
			35.222 °C	32.705 a	29.110 a	34.595 b	0.700 c
SEM				0.427	0.197	0.442	0.058
*p* values							
Additive				0.042	0.479	0.078	0.018
Usage				0.003	0.030	0.086	0.026
Temperature				0.000	0.000	0.000	0.000
Additive × Usage				0.064	0.131	0.328	0.055
Additive × Temperature				0.826	0.302	0.218	0.000
Usage × Temperature				0.688	0.762	0.487	0.034
Additive × Usage × Temperature				0.565	0.224	0.967	0.702

a–d: Values shown in different letters in the same column are statistically important (*p* < 0.05). * Storage temperatures were recorded by data logger on day 4, while thermal camera images were taken. AV: Apple vinegar; SDA: Sodium diacetate; Std. Dev.: Standard deviation; and SEM: Standard error of means.

**Table 8:** Thermal camera imaging results of aerobic stability on day 7 (Ambient temperature = 22 °C).

Aerobic Stability	Additive	%	Storage Temperature *	Thermal Camera Imaging Measurements, °C			
				Mean	Min	Max	Std. Dev.
Day 0	-	-	-	30.62	26.75	33.91	0.94
Day 7	AV	0	28.060 °C	30.665 def	27.780 bc	32.345 cde	0.685 cde
			35.971 °C	34.290 a	29.565 a	36.265 a	1.155 ab
		0.5	28.060 °C	31.535 cde	27.875 bc	33.580 bcd	0.880 bcd
			35.971 °C	33.800 a	29.235 ab	35.890 a	1.050 ab
		1.0	28.060 °C	30.520 def	27.505 c	32.515 cde	0.565 de
			35.971 °C	32.670 abc	28.050 bc	35.110 ab	1.105 ab
	SDA	0	28.060 °C	29.265 f	27.250 c	31.375 e	0.535 e
			35.971 °C	32.895 abc	29.080 ab	35.015 ab	0.970 abc
		0.5	28.060 °C	31.925 bcd	28.485 abc	34.440 abc	0.925 bc
			35.971 °C	33.430 ab	28.440 abc	36.075 a	1.295 a
		1.0	28.060 °C	30.115 ef	27.425 c	32.205 de	0.690 cde
			35.971 °C	33.280 abc	29.075 ab	35.450 ab	0.995 abc
SEM				0.340	0.180	0.369	0.052
*p* values							
Additive				0.178	0.867	0.622	0.933
Usage				0.032	0.262	0.033	0.023
Temperature				0.000	0.000	0.000	0.000
Additive × Usage				0.113	0.306	0.235	0.131
Additive × Temperature				0.887	0.867	0.895	0.844
Usage × Temperature				0.098	0.204	0.189	0.416
Additive × Usage × Temperature				0.499	0.160	0.767	0.344

a–f: Values shown in different letters in the same column are statistically important (*p* < 0.05). * Storage temperatures were recorded by data logger on day 7, while thermal camera images were taken. AV: Apple vinegar; SDA: Sodium diacetate; Std. Dev.: Standard deviation; and SEM: Standard error of means.

**Table 9:** Thermal camera imaging results of aerobic stability on day 12 (Ambient temperature = 22 °C).

Aerobic Stability	Additive	%	Storage Temperature *	Thermal Camera Imaging Measurements, °C			
				Mean	Min	Max	Std. Dev.
Day 0	-	-	-	30.62	26.75	33.91	0.94
Day 12	AV	0	27.567 °C	28.770 c	26.840 bc	30.360 c	0.415 d
			36.295 °C	32.610 a	28.875 a	34.685 a	0.815 abc
		0.5	27.567 °C	27.645 c	25.925 c	29.720 c	0.470 d
			36.295 °C	33.310 a	29.190 a	35.250 a	0.945 a
		1.0	27.567 °C	27.710 c	26.170 bc	30.000 c	0.455 d
			36.295 °C	32.790 a	28.955 a	35.050 a	0.855 abc
	SDA	0	27.567 °C	30.010 b	26.125 bc	32.285 b	0.840 abc
			36.295 °C	32.895 a	29.185 a	35.565 a	0.925 a
		0.5	27.567 °C	29.925 b	26.905 bc	32.080 b	0.695 bc
			36.295 °C	32.810 a	28.920 a	34.940 a	0.890 ab
		1.0	27.567 °C	30.260 b	27.170 b	32.045 b	0.665 c
			36.295 °C	33.325 a	29.880 a	35.315 a	0.830 abc
SEM				0.445	0.297	0.460	0.040
*p* values							
Additive				0.000	0.086	0.000	0.001
Usage				0.844	0.394	0.705	0.458
Temperature				0.000	0.000	0.000	0.000
Additive × Usage				0.304	0.095	0.777	0.095
Additive × Temperature				0.001	0.805	0.001	0.002
Usage × Temperature				0.219	0.919	0.724	0.574
Additive × Usage × Temperature				0.246	0.105	0.345	0.898

a–d: Values shown in different letters in the same column are statistically important (*p* < 0.05). * Storage temperatures were recorded by data logger on day 12, while thermal camera images were taken. AV: Apple vinegar; SDA: Sodium diacetate; Std. Dev.: Standard deviation; and SEM: Standard error of means.

## Data Availability

The data that support the findings of this study are available from the corresponding author, upon reasonable request.
